# The economy of chromosomal distances in bacterial gene regulation

**DOI:** 10.1038/s41540-021-00209-2

**Published:** 2021-12-15

**Authors:** Eda Cakir, Annick Lesne, Marc-Thorsten Hütt

**Affiliations:** 1 Department of Mathematics and Logistics, Jacobs University, D-28759 Bremen, Germany; 2 Department of Life Sciences and Chemistry, Jacobs University, D-28759 Bremen, Germany; 3 Institut de Génétique Moléculaire de Montpellier, University of Montpellier, CNRS, F-34293 Montpellier, France; 4 Sorbonne Université, CNRS, Laboratoire de Physique Théorique de la Matière Condensée, LPTMC, F-75252 Paris, France

**Keywords:** Regulatory networks, Complex networks

## Abstract

In the transcriptional regulatory network (TRN) of a bacterium, the nodes are genes and a directed edge represents the action of a transcription factor (TF), encoded by the source gene, on the target gene. It is a condensed representation of a large number of biological observations and facts. Nonrandom features of the network are structural evidence of requirements for a reliable systemic function. For the bacterium *Escherichia coli* we here investigate the (Euclidean) distances covered by the edges in the TRN when its nodes are embedded in the real space of the circular chromosome. Our work is motivated by ’wiring economy’ research in Computational Neuroscience and starts from two contradictory hypotheses: (1) TFs are predominantly employed for long-distance regulation, while local regulation is exerted by chromosomal structure, locally coordinated by the action of structural proteins. Hence long distances should often occur. (2) A large distance between the regulator gene and its target requires a higher expression level of the regulator gene due to longer reaching times and ensuing increased degradation (proteolysis) of the TF and hence will be evolutionarily reduced. Our analysis supports the latter hypothesis.

## Introduction

Approaches from Systems Biology have led to remarkable progress in understanding bacterial gene regulation^1–3^. Instrumental in this progress is the formal representation of gene regulation as a network of gene-gene interactions mediated by TFs, which allowed identifying some design principles underlying this class of biological processes. Among these are the role of small regulatory devices like coherent feedforward loops ensuring noise buffering^4^, feedback loops and incoherent feedforward loops implementing adaptation to long-term stimulation^1,5^, groups of genes under a common regulation as a suitable structure to run temporal programs^6,7^, as well as the functional relevance of a hierarchical organization of the interactions^8^.

One of the remarkable conceptual approaches put forward by network science is the possibility to ask in a systematic fashion about the nonrandom features of a given network. In this way, diverse systems can be compared on a quantitative level using unified statistical tools. Provided a suitable null model is used, such nonrandom features can often be associated with functional requirements of the network and/or an optimization installed in the network by some evolutionary pressure. It was for instance the statistical observation of a high abundance of feedforward loops in gene regulatory networks^6^ that preceded their mechanistic interpretation as noise-buffering devices in the coherent case and ’pulse generators’ in the incoherent case.

However, we are far from a comprehensive understanding of bacterial gene regulation. In particular accumulating evidence points to the need of considering the regulatory network as a spatially embedded structure, where the spatial organization of the circular bacterial chromosome contributes to the overall regulation of genes^9–13^. This challenge, among others, has to date prevented for example the creation of predictive models of bacterial gene expression patterns and the achievement of a mechanistic understanding of bacterial gene regulation. Relating the network representation with the chromosomal organization in real space is a decisive step along this way.

**Fig. 1:**
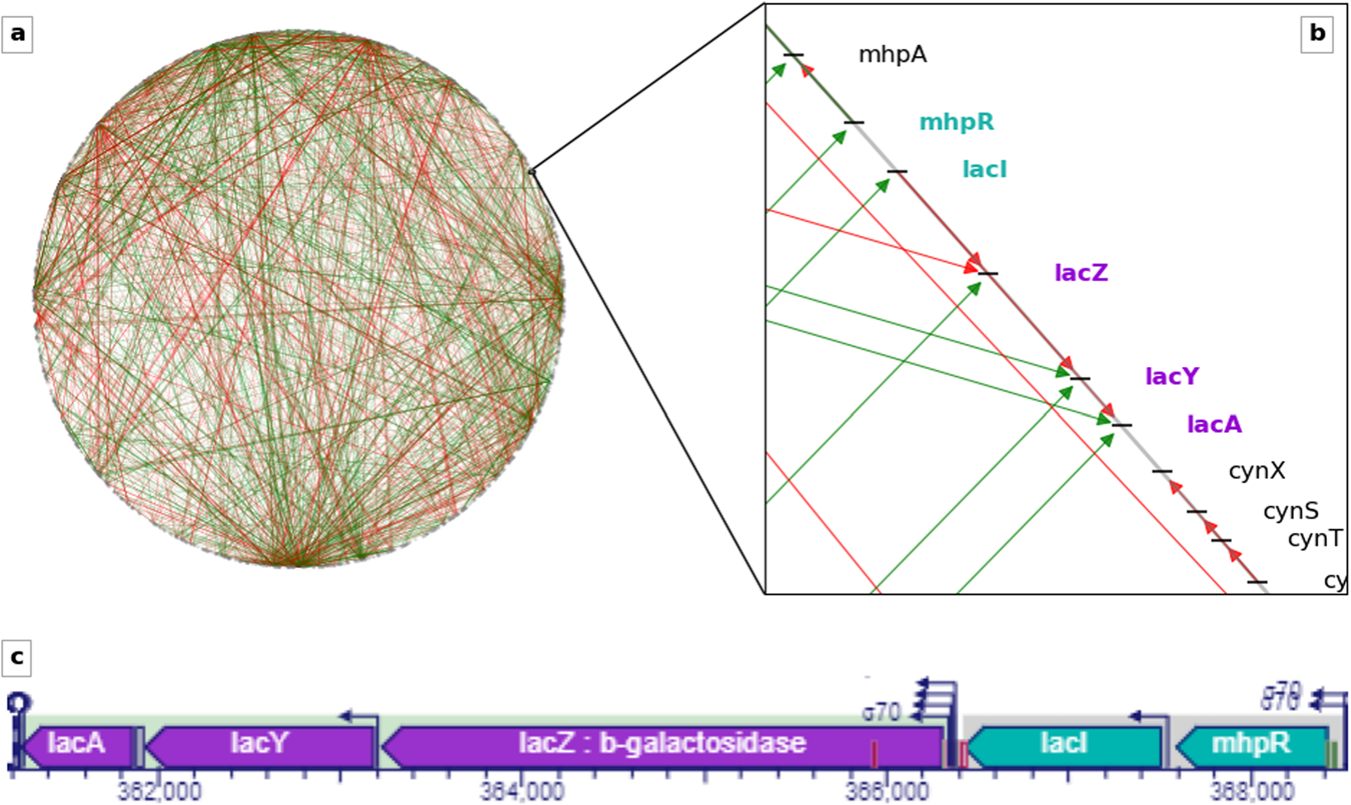
Transcriptional regulatory network (TRN) of *E. coli*. **a** Via the chromosomal coordinates of each gene, the TRN can be represented as embedded in a circular space given by the bacterial chromosome. Nodes represent genes. Blue, red, grey directed edges from the regulator genes to the target genes represent the action of activating, repressing, dual/unknown transcription factors (TFs), respectively. **b** Magnified view of one spatial region containing the operons lacZYA and mhpR-lacI (gene name colors represent operon membership). **c** Standard EcoCyc^50^ visualisation of the operons highlighted in (**b**).

The fact that the transcriptional regulatory network (TRN) needs to be considered as a spatially embedded network is particularly clear for a bacterium, as there transcription and translation are not localized in different cellular compartments. This allows us to leverage methods from network science, designed for the analysis of spatially embedded networks, for an even further-reaching statistical investigation of these nonrandom features^14,15^. Figure 1 introduces this view of the TRN in its chromosomal embedding.

The pioneering work by Warren and ten Wolde^16^ first studied the spatial embedding of the transcriptional regulatory network and interpreted some of its nonrandom features from a biological perspective. In addition to this early work, a lot is already known about the nonrandom features of this spatially embedded network. Regulated and non-regulated genes have markedly different statistical distributions along chromosome^17^. Genomic distances among target genes of the same TF are smaller than expected at random^16^. The chromosomal axis defined by the origin of replication and the terminus of replication (Ori-Ter axis) is an important organizer of gene activity^12,13^ and also shows up in statistical properties of the TRN^13^. In contrast to other spatially embedded networks, the TRN does not show a systematic decrease of link density with genomic distance^15^. Genomic neighborhood and TRN explain gene expression patterns in a complementary fashion, suggesting a buffering mechanism between two types of regulation, one related to the TRN and the other to chromosomal structure^11^. We here extend this line of investigation by studying the interplay of network features and spatial organization and their correlation with gene expression levels.

Direct imaging of the *E. coli* chromosome shows a circular structure^18^ that shades the view of a highly condensed nucleoid^19^. Local heterogeneities are observed along this circle. Its circular shape fluctuates in time and from cell to cell, however with a variation of not more than 30% in width and length (perimeter), supporting an average picture of a circular chromosome^18^. We can expect the position of genes in the circular chromosome to be evolutionarily optimized. One functional aspect to this positioning of genes is the implementation of temporal programs via the order of genes locally on chromosome^12^. This is apparent in the organization of groups of genes in operons (cluster of a few adjacent genes contributing to the same biological function) and other aspects of the clustering of genes on smaller or larger chromosomal scales^16,20–23^.

A still unexplored level of evolutionary optimization is the wiring economy of the TRN. The hypothesis of a parsimonious usage of ’wiring’ (spatial distances along edges) in a network, due to construction, maintenance, and (signal) transportation costs, has been intensely discussed in Neuroscience, where the notion of ’wiring economy’, i.e., the minimization of the total wiring length (i.e., the wiring cost) with regard to the signal transportation efficiency, has been introduced^24–27^.

In order to assess the wiring economy of the TRN, we resort to methods developed and applied in the context of the cellular network formed for nutrient transportation by the slime mould *Physarum polycephalum*^28^ and brain networks on the level of cortical areas^27^. This is achieved by addressing the statistical question, whether the distances in space spanned by the edges of the network (often referred to as the ’wiring’ of the network) are typically larger or smaller than expected at random. We also compare these observations with the signaling capabilities of the network (below defined as ’regulatory span’) and discuss the biological implications of these observations (e.g., how these nonrandom features will be reflected in gene expression data). We define the regulatory span as the percentage of nodes directly or indirectly reached by each TF. One might formulate refined versions of this regulatory span, e.g., by weighting direct and indirect targets differently.

Two distinct hypotheses can be formulated:

1. The local regulation of genes takes place via chromosomal structure, as determined by the distribution of supercoiling energy along the chromosome^29,30^ and locally stabilized by the binding of structural proteins or nucleoid-associated proteins (NAPs)^9,31^. This hypothesis suggests that dedicated TFs are more likely to be associated with long-distance regulation, rather than short-distance regulation, leading to the expectation of *high* total wiring length (i.e., *low* wiring economy).
2. The limiting factor for the regulation of genes via TFs is the cost of producing sufficient numbers of each individual TF to reach its targets (in spite of the dilution due to spatial diffusion and the possibly long-reaching time, entailing a risk of proteolysis of the TFs), thus favoring more proximal targets and hence the expectation of *low* total wiring length (i.e., *high* wiring economy). At the same time, this hypothesis—due to the hypothesized evolutionary pressure on the number of produced TFs—would also suggest a discernible correlation between distance and gene expression level.

Here we test these two broad, general pictures of the wiring economy of bacterial gene regulation against data for the bacterium *E. coli*.

## Results

### Wiring economy and processing steps

As a first step, we investigate, whether the spatial distances covered by the edges of the network (wiring lengths) and the average number of processing steps from source nodes (regulators) to target nodes (regulated genes) are larger or smaller than expected at random.

**Fig. 2:**
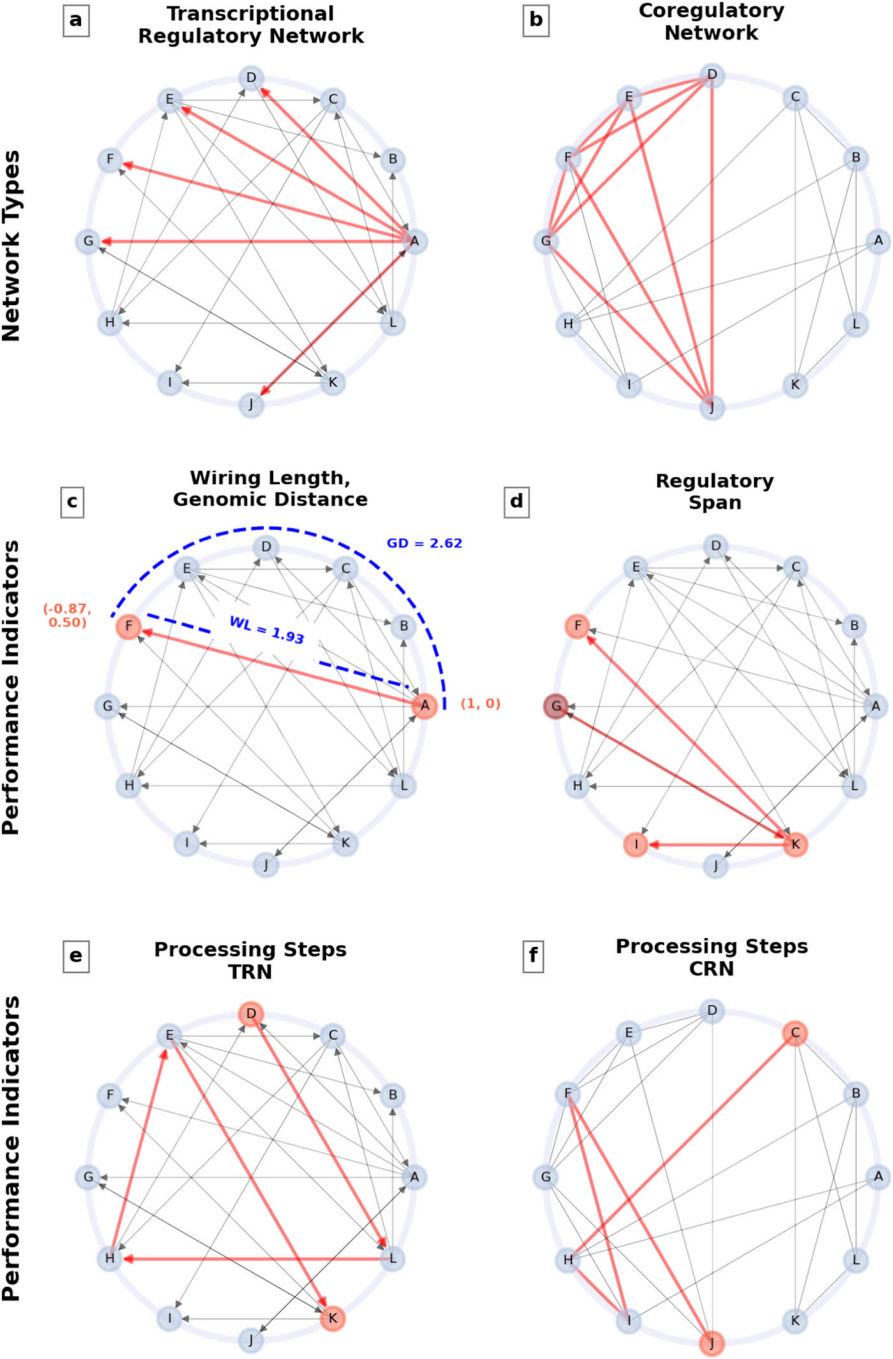
Schematic representation of the main quantities of our investigation. **a** TRN. Directed edges represent the regulatory action of the TF encoded by the regulator gene/operon, pointing to the regulated gene/operon. Red edges highlight the regulation of nodes D, E, F, G, and J by node A. **b** CRN. Nodes are the genes/operons, as in the TRN, but edges connect pairs of genes/operons coregulated by a third gene/operon. Nodes D, E, F, G, and J are all connected (red edges) due to their joint regulation by node A. **c** Wiring length (WL) and Genomic distance (GD). The wiring length of the edge from node A to F corresponds to the spatial (Euclidean) distance between the centers of the nodes A and F (WL = 1.93). For each pair of connected nodes, we also consider the arc length between the centers of the nodes, i.e. genomic distance (GD = 2.62). **d** Regulatory span of node G. **e, f** Number of processing steps in the TRN (**e**) and the CRN (**f**). The number of processing steps is the average number of steps along the shortest path between nodes. In the TRN, for the node pair (D, K), the number of steps along the shortest path D-L-H-E-K is 4. In the CRN nodes C and J are linked via four processing steps. This number is 1 for node pairs related by an edge (direct neighbors).

We perform our analysis on the TRN and the coregulatory network (CRN, i.e., the network where nodes are the genes/operons and edges are the links between gene/operon pairs regulated by a common transcription factor; see Methods) of E. coli (see Fig. 2a, b). To analyze the TRN and the CRN both on gene and operon levels, we employ three TRN and four CRN null models (see Methods) and generate 1000 random networks per each null model. While the node swap and the random node position methods randomize spatial distances, but do not alter the network (and hence retain the number of processing steps) both edge swap methods discard these features and randomize source-target node pairs.

**Fig. 3:**
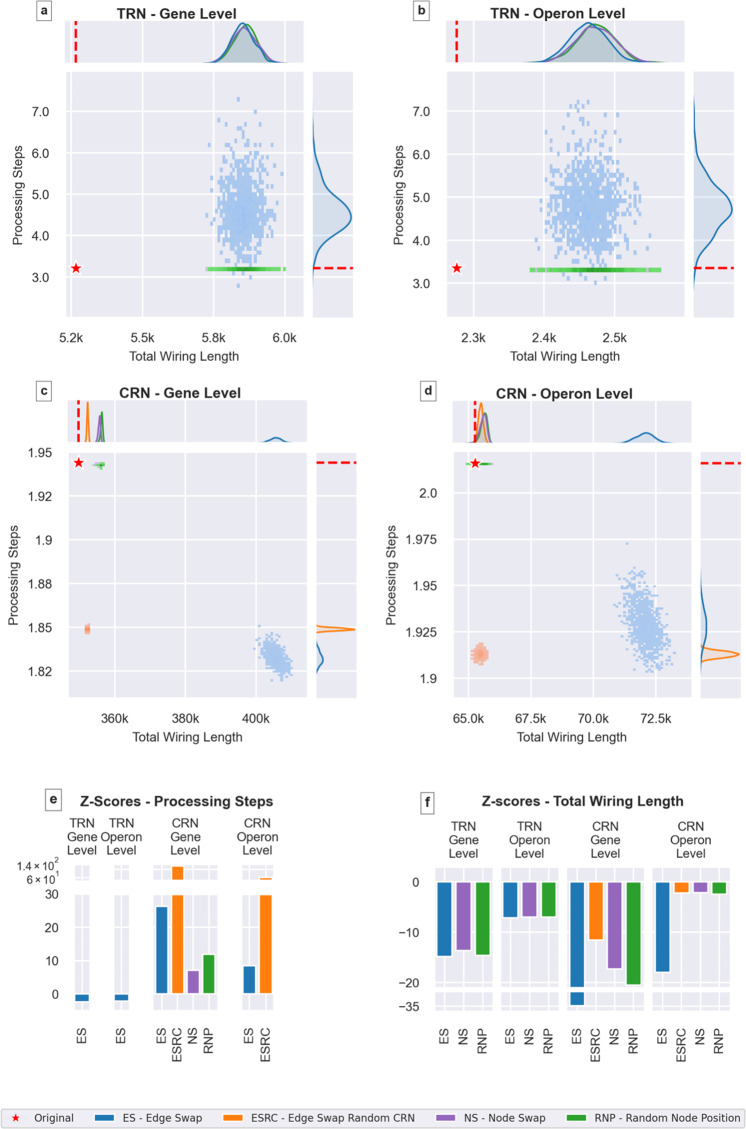
Comparison of the original network () and null models (dots colored according to null model type) in terms of wiring length and number of processing steps in the TRN (a,b) and the CRN (c,d) on the gene (a,c) and operon (b,d) levels. **a** Gene-level TRN. Original network has a significantly lower wiring length than the randomized networks with an average z-score of − 14.38. The number of processing steps is also lower in the original network, compared to its randomized counterparts (z-score =−2.37). **b** Operon-level TRN. Results are consistent with the gene level (wiring length average z-score =−7.05, processing steps z-score =−2.20). **c** Gene-level CRN. The original network has significantly lower wiring length than the randomized networks with an average z-score of−21.05. The number of processing steps is much higher than expected at random (average z-score = 42.15). **d** Operon-level CRN. Results are consistent with the gene level (wiring length average z-score =−6.21, processing steps average z-score = 17.44). **e** Processing step z-scores. **f** Wiring length z-scores.

As seen in Figs. 3a and 3e, the wiring lengths of the original (gene-level and operon-level) TRNs are significantly smaller than the wiring lengths of the generated randomized networks (with z-scores between − 14.86 and − 13.68 on the gene level). Even though source-target node pairs (edges) or node positions are randomized via three different methods, we observe similar wiring length distributions for each null model, which indicates that both source-target pairs and node positions, i.e., both the association between regulators and regulated genes and their positions, play an important role in improving wiring economy.

We investigate this nonrandom property of the embedded network also by performing all the analyses using genomic distance instead of wiring length (see Methods) to account for one-dimensional sliding of TFs along the DNA. The results are consistent with the observation in Fig. 3, showing that regardless of the TF’s target ’search method’, the network displays high wiring economy (Supplementary Figure 2).

It is conceivable that setting constraints in numerically generating networks to prevent self-regulation may create a bias towards higher wiring length at randomized networks (the elimination of interactions that originate and end at the same operon, see Methods, Section 4.1). We studied the impact of this constraint and found no significant impact on wiring lengths (Supplementary Figure 3).

The real CRN also has a significantly lower wiring length than randomized networks with z-scores between − 34.88 and − 11.53. The null model CRNs derived from randomized TRNs (see Methods) have the highest wiring lengths (Fig. 3c, d). Since the source and target node pairs are not conserved and the edges are randomized by swapping, network properties like modularity are not preserved in this method. For these null model CRNs, the average number of edges is 14.5% higher than in the real CRN (and, by construction, the other null model CRNs). This supports the view that coregulated genes tend to lie closer to each other and coregulated gene subgroups tend to be regulated by the same regulators. Destroying this clustering on the TRN level results in increased variability of coregulated gene subgroups and an increase in the number of edges in CRN. From a biological perspective, the choice of this null model (CRNs derived from randomized TRNs, rather than directly randomized CRNs) is motivated by the view that the CRN is rather a relational structure, while the actual biological ’hardware’ resides in the TRN.

These results are confirmed by analyzing networks on the operon level (z-scores between − 7.15 and − 6.98). As in Warren et al. ^16^, our analyses show that pairs of operons that are connected in TRN tend to be closer to each other than expected at random.

In terms of processing steps, Fig. 3 shows that the average number of steps required to reach target nodes is less in the real TRN than in randomized networks (z-scores are − 2.37 and − 2.20 at gene and operon level respectively). However, the regulatory span, i.e., the percentage of nodes (directly or indirectly) reachable from a source node, is also relatively low. While 28.2% of the nodes are reachable on average in the (gene-level) randomized networks, the regulatory span for the real (gene-level) TRN is at 10.4% (Supplementary Figure 4).

By going beyond direct links, the number of processing steps, as well as the regulatory span reveal, to what extent a transcription factor (source node) potentially affects systemic components further downstream of the direct regulation. This comparison of number of processing steps and regulatory span suggests a clear overall picture: In order to ensure a lean and efficient network, the number of descendants is low, but the number of processing steps is enhanced.

The number of processing steps of the real CRN is higher than expected at random (Fig. 3c, d). However, it should be noted that the TRN-level randomization does not conserve the number of edges in the randomized CRNs.

Summarizing these observations, the TRN—the ’hardware’ implementing regulation—unites efficient processing (lower-than-random average number of processing steps), very efficient wiring (much lower-than-random total wiring length) at the expense of parallelized information distribution (lower-than-random regulatory span). The CRN—the structure underlying coherent activity patterns—is spatially compact (lower-than-average wiring), but shows less efficient processing (higher-than-random average number of processing steps), suggesting the possibility to decouple sub-patterns of activity.

### Interpretation and functional significance

The effect of distance on the efficiency and reliability of transcriptional regulation has long been studied. Since the work by Riggs et al.^32^ and Berg et al.^33^, it is acknowledged that proteins could find their target sites via a combination of one-dimensional sliding along the DNA and three-dimensional diffusion through the cytoplasm. It is also known that for the 1D component of the search process, the search time of a TF can depend on the initial position of the TF, i.e., the position of the regulator gene^34^. The average search time is estimated to be faster if a TF could find its target site via 1D sliding (≈0.3 sec) rather than a combination of 1D sliding and 3D diffusion (≈150 min)^21^. Moreover, degradation of TFs (proteolysis) is expectedly present (such degradation is unavoidable, as mechanisms for setting the system back to a default state and adapting the regulation by TFs to different situations). Assuming a first-order kinetics, the degradation is exponential with time.

The distance between the regulator gene and the regulated gene affects the speed and reliability of transcriptional regulation in bacterial cells substantially^35^. Efficiency and consistency of gene regulation depend on how close the regulator gene is to the site on DNA the TF has to bind, i.e., the promoter region of the regulated gene^21^. Pulkkinen and Metzler showed in their study^35^ that the effect of the distance on the regulation efficiency is significant, i.e., the shorter the distance, the stronger and faster the response. It was also claimed that for efficient gene regulation, the TF concentrations should be high, and the high TF concentrations can be reached through gene proximity. These constraints promote the colocalization of the regulator genes and their targets on the genome. Similarly, at the operon level, the fact that the coregulated operons tend to colocalize is also highlighted by Warren and ten Wolde^16^ by comparing the network with randomly created ones.

Prompted by this collection of indirect evidence relating source-target distances with gene expression and by our result of a strong preference in the TRN of short source-target distances, we now analyze the rate of gene transcription, which provides a measure of the network transcriptional regulation capacity, and its relationship with the spatial distance between the regulator and regulated genes by investigating the correlation between the TRN wiring economy and the expression levels of TFs. We use a highly structured RNA-seq dataset, which contains 278 gene expression profiles^36^.

**Fig. 4:**
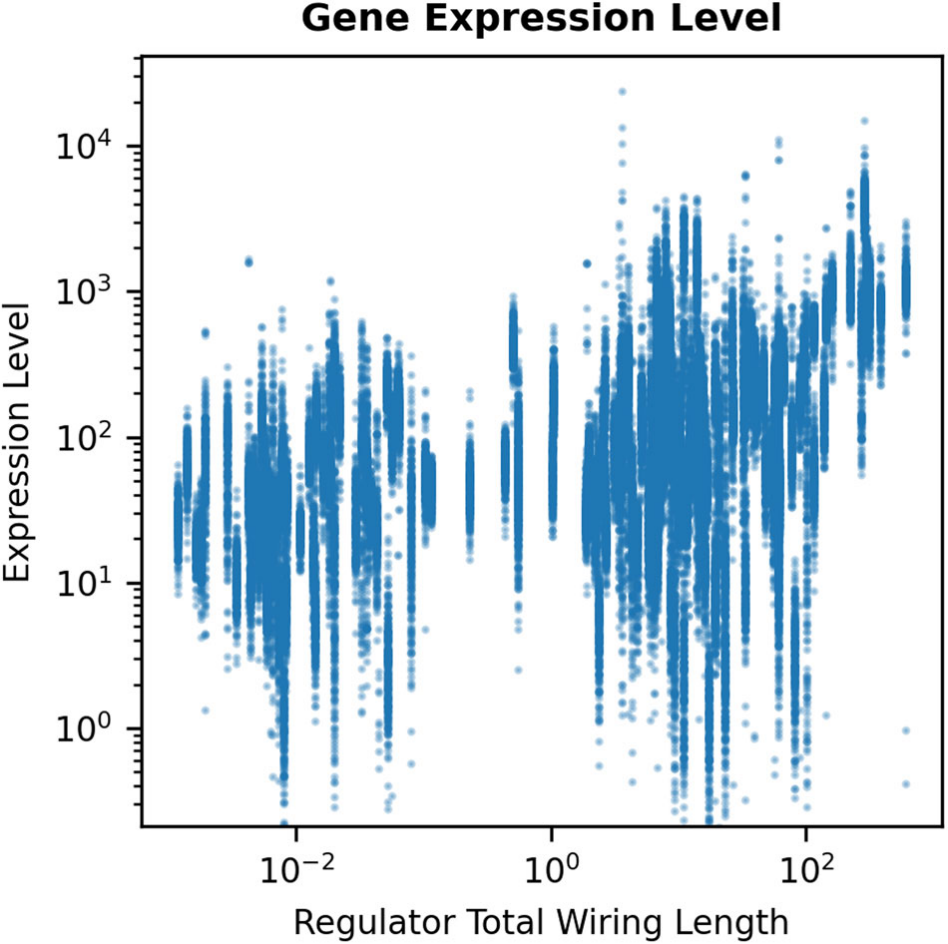
Scatterplot of the expression levels of the regulator genes as a function of total wiring length of outgoing edges from each regulator gene to all its target nodes (regulator total wiring length). The gene expression levels positively correlate with the regulator total wiring lengths (average Spearman correlation across all RNA-seq datasets (see Methods): 0.4165; average *p* value: 1.31 × 10^−6^; maximal *p* value: 1.18 × 10^−4^).

We investigate the possible relationship between wiring length and the expression level of the regulator genes (source genes in the TRN). The total wiring length of the outgoing edges from each regulator and the expression level of each regulator are found to be significantly correlated (Fig. 4, Spearman correlation coefficient: average = 0.4165, *p* values of all profiles: < 1.18 × 10^−4^, average = 1.31 × 10^−6^). Considering the significant correlation, it can be argued that with the effect of limiting noise and increasing reliability, the wiring economy is enhanced. Long-distance and correspondingly long wiring length are associated with an increase in gene expression level.

As with many statistical associations of dynamical data (here: gene expression data) and network structure (here: the spatially embedded TRN), it is not possible to disentangle the various contributions. Correlation between expression levels and total wiring length is highest among the quantities we analyzed, but also (in descending order) maximal wiring length, the out-degree, and the average wiring length show highly significant positive correlations with gene expression levels (see Supplementary Table 1), with the maximal wiring length being, for a given regulator, the maximal spatial distance to its target genes.

Gene expression data, at present, do not allow for such detailed assessment (which could, however, be envisioned for single-cell measurements), but it is nevertheless informative to look in more detail at the relationship between the gene expression levels and distances expected from general considerations. The object relating these two quantities is the reaching time of a transcription factor with respect to its target site. A starting point for a corresponding theoretical framework is outlined in Supplementary Text 1.

As an application of our spatially embedded view of the bacterial gene regulatory network, we use our framework to disentangle the biological nature and function of two related categories: the standard *Regulons*^37^ and the recently introduced *iModulons*^36^. Regulons are defined as groups of genes regulated by one regulator^37^. The concept of iModulons denotes gene sets that represent independently modulated signal processing units derived by applying independent component analysis to RNA-seq datasets. Genes are grouped into such sets by observing patterns in the transcriptome expression data. Specifically, iModulon detection involves blind source separation, i.e., the separation of environmental conditions and internal regulation via unsupervised machine learning.

**Fig. 5:**
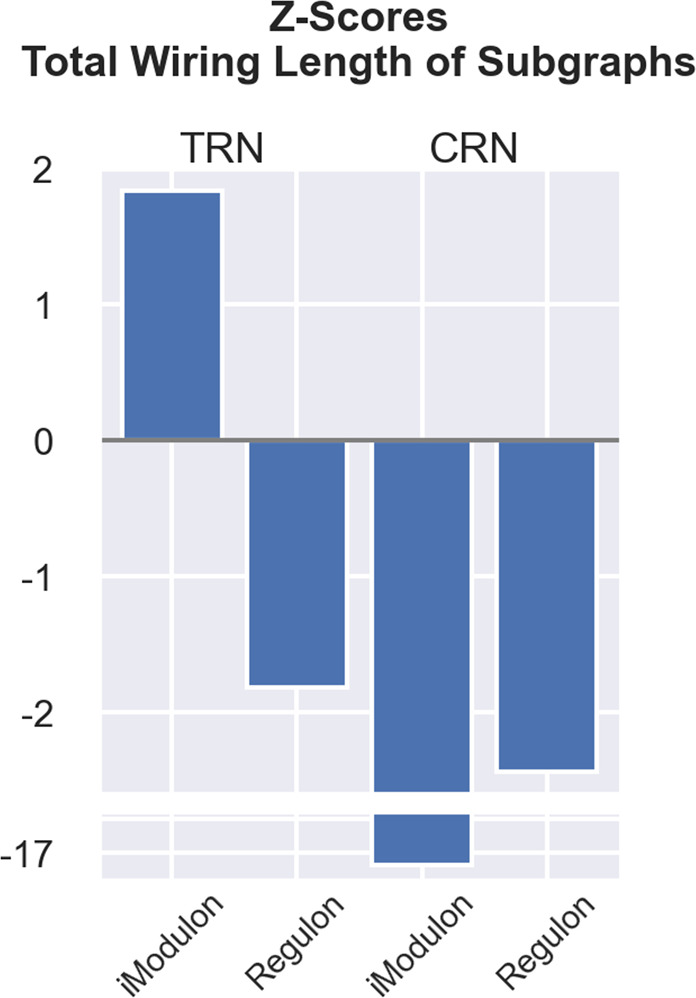
Comparison of total wiring lengths for iModulon and Regulon subgraphs in the TRN and CRN. We calculate the wiring lengths of iModulon/Regulon subgraphs as the sum of wiring lengths for all edges between the nodes in the subgraphs, ensuring that the main regulators are in the considered subgraphs (The main regulator(s) is added to the subgraph if it is not one of the nodes in the considered subgraph.). The total wiring length is the sum of the wiring lengths of all iModulon/Regulon subgraphs in the TRN/CRN. For the z-scores listed here, we employ the network compiled in the original iModulon publication^36^ and generate 1000 randomized networks using the node swap method (see Methods).

In Sastry et al.^36^ the authors find that the iModulons are similar to, but distinct from, Regulons. Around 66% of the identified iModulons have significant overlaps with Regulons. Here we show that the embedded-network perspective allows us to uncover different organizational principles behind these two types of regulatory units in bacterial gene regulation. In order to evaluate iModulons from the perspective of embedded networks, we employ the (larger) network compiled in the original iModulon publication^36^, rather than the standard RegulonDB network from ref. ^38^. Figure 5 summarizes the results.

Our analysis shows that from a regulatory perspective (i.e., evaluating the spatial embedding of the TRN), iModulons are unspecific in their spatial organization, while Regulons are spatially localized. In the case of coregulation (i.e., evaluating the spatial embedding of the CRN and thus assessing the capacity to generate coherent activity patterns) both units are spatially tightly clustered (i.e., their average wiring length is much shorter than expected at random). These nonrandom features of Regulons and iModulons are further evidence of the involvement of space in the organization of bacterial gene regulation. Furthermore, the embedded-network analysis reveals a discriminating feature between the two types of regulatory units: Regulons are spatially compact in regulation (as evidenced by the shorter than-random wiring length of the corresponding TRN subgraphs) and compact in co-activation (as indicated by the shorter-than-random wiring length of the corresponding CRN subgraphs). On the other hand, iModulons show a low wiring economy in regulation, but a substantial wiring economy in co-activation.

## Discussion

Several technicalities make this investigation of TRNs challenging: (1) It is not clear, whether this statistical analysis should be performed on the level of genes or the level of operons. We do both and find that the results are quite similar, suggesting that the organization of bacterial genes into operons is not directly affecting the wiring economy of these networks. (2) It is not clear, whether the relevant network for the discussion of wiring economy is the TRN with its regulatory interactions or rather the CRN with its links representing associations of genes due to common regulation. Again we perform our analysis on both networks.

A limitation of our analysis is that even in the case of a well-investigated model organism, like E. coli, our knowledge about transcriptional regulation is incomplete. In fact, current estimates in RegulonDB^38^ assume that substantial parts of the network are still missing. We can only address this limitation by continuing to monitoring the features analyzed here in future versions of the TRN, when they appear (see, e.g., ref. ^39^).

A crucial step in such an investigation is the choice of the null model. A null model here is the ensemble of random graphs the original network is contrasted with. A null model is characterized by the set of network properties it preserves. Clearly, when the null model is too different from the original network, then almost any network property will seem nonrandom. One therefore needs to be very explicit in the construction of the null model, which features of the original network are preserved and which are randomized. In addition, discarding through randomization different features of the original network can provide further insight into the driving mechanisms behind the identified nonrandom features.

Here we opted for three null models: (1) Degree-preserving switch randomization. This method has been developed in ref. ^40^ and employed in ref. ^6^ and ref. ^41^. Even though the exact degree sequence of the graph is preserved, higher-order network properties like modularity are destroyed. When assessing for example the small subgraph distribution of a random modular graph with standard switch randomization as a null model, an artifactual nonrandom distribution of small subgraphs would be observed^42^. (2) Assigning random chromosomal positions to all genes without altering the network. This null model is particularly relevant for the assessment of wiring economy, as the distances covered by each link in the network are efficiently randomized. However, this particular null model does not preserve the highly nonuniform gene density across the circular chromosome. (3) Randomly positioning genes, but retaining the original list of gene positions (iterative gene swapping). In this case, both the network and gene density are conserved, but distances are still randomized.

We explored a fundamental principle of bacterial gene regulation, namely its spatial embedding. For both the transcriptional regulatory network (TRN) and the coregulatory network (CRN), we investigated their wiring economy, a concept popular in the analysis of neural connectivity patterns. We find high wiring economy (shorter than-random spatial distances of regulatory interactions) in the transcriptional regulatory network of the bacterium E. coli, suggesting an evolutionary pressure to avoid long-distance regulation. We can hypothesize that this evolutionary pressure is due to the cost of producing mRNAs, as high mRNA levels are required to diffusively reach distal regulatory targets. This interpretation is confirmed by the scaling of average transcriptome levels (as measured in RNA-seq experiments) with the distance between the source and target genes in the TRN. Future works could take benefit from single-cell RNA sequencing in situ that are developed and will soon be routinely available^43^.

Our findings suggest that gene expression levels need to be corrected for source-target distances before functional interpretation. This can have implications for the statistical assessment of differentially expressed genes and for network inference algorithms operating on transcriptome profiles.

Our two initial hypotheses do not conflict with each other. Even though our results show strong support for one hypothesis, the other hypothesis (enhanced long-distance regulation by TFs) may also be valid while being just masked by the much stronger nonrandom feature of high wiring economy. Detecting such nonrandom features on multiple scales would require a careful statistical assessment of the whole probability distribution of genomic distances between a TF and its target genes. This calls for a new class of null models capable of preserving the nonrandom network features on one scale, in order to quantify nonrandom features on a different scale.

The results presented here can serve as a starting point for a range of further investigations: validating these findings in other bacterial systems, a step currently impeded by the lack of detailed, high-quality data on TRNs in other organisms; extension to eukaryotic organisms (e.g., yeast, for which some information on the TRN is available^44^), where the spatial organization is far more complex and its regulatory contribution, though undisputed, is hard to quantitatively assess.

When high-resolution single-cell data of chromosome structure and chromosome dynamics will become available, one can start exploring these questions beyond the circle approximation employed here, in order to study the interplay of chromosomal dynamics and TF-based gene regulation in further detail^45,46^.

On the more theoretical side, a direction of future work is to investigate the robustness of the wiring economy discovered here with respect to random fluctuations of chromosomal organization.

## Methods

### Transcriptional regulatory network (TRN) and coregulatory network (CRN)

The statistical analysis of the TRN of the bacterium E. coli is performed considering the TRN as a simple directed graph, either on the gene or the operon level. The nodes of the constructed networks represent genes (gene level) or operons (operon level) and directed edges represent the transcriptional regulation between these nodes. In the gene level TRN, for instance, since the gene *acrR* encodes the protein *AcrR* to regulate the gene *acrB*, there is a directed edge from node acrR to node acrB. In the operon level TRN, since the gene *acrR* in the operon *acrR* encodes the protein *AcrR* and regulates the gene *acrB* in the operon *acrAB*, there is a directed edge from the operon *acrR* to the operon *acrAB*.

We compile the TRN using the dataset from RegulonDB v10.5^38^, a comprehensive database that provides information on transcriptional regulations of E. coli K-12. To create a directed simple graph, multiple interactions between the nodes and the interactions originating and ending in the same operon (both, for the gene-level and the operon-level TRN) are removed from the network. The resulting TRNs consist of 4601 interactions between 1841 genes on the gene level and 1942 interactions between 910 operons on the operon level.

We assume that the chromosome is circularly embedded in 2D space. The polar coordinates of the nodes on a unit circle are calculated using the positions of the genes along the chromosome in terms of base pairs.

We also perform our analysis on the CRN of E. coli on gene and operon levels. In the CRN, nodes are the genes/operons, as in the TRN, but edges are the links between pairs of genes/operons coregulated by a third gene/operon. The simple undirected coregulatory graph is the representation of the coregulated pairs. Such a CRN visualizes the capacity of connected units to display a coherent pattern of activity. A statistical comparison of the CRN and TRN representations of the gene regulatory system has been, for example, performed in ref. ^47^. Figure 2a, b illustrate these two network types in a schematic fashion.

We use the NetworkX Python package for the creation and manipulation of the regulatory networks^48^.

### Performance indicators: wiring length, genomic distance, number of processing steps, and regulatory span

Considering the chromosome to be circularly embedded in 2D space, the spatial (Euclidean) distance between the centers of the nodes, i.e., *wiring length*, serves as a proxy for the time spent in diffusing through the cytoplasm. One-dimensional sliding along the DNA is investigated by calculating the arc length between the centers of the nodes, i.e., *genomic distance*.

The total wiring length is computed as the sum of wiring lengths for all edges in the network. The same applies to the total genomic distance. Note that in contrast to the chromatin organization in eukaryotic organisms, here the genomic distance (along the circular chromosome) and the spatial distance (in space) are related: A high genomic distance implies a high spatial distance.

In a simple directed graph (TRN), the average number of steps along the shortest paths for all source and target node pairs is denoted as the number of *processing steps*. Regarding CRNs (simple undirected graphs), the average number of steps is defined as the average number of steps of the largest connected component. A schematic illustration of these performance indicators is given in Fig. 2c, d.

Another measure to evaluate graph connectivity is the *regulatory span*. Regulatory span is the average number of reachable nodes from regulators (directly or indirectly) compared to the total number of nodes in a network. Note we do not compute the regulatory span for the CRN, as it is not a meaningful quantity there, due to the undirected nature of this network.

### Null models

We employ the standard switch randomization algorithm^40^, i.e., edge swapping, to create uniformly distributed directed random networks preserving in- and out-degrees. We keep the node positions fixed and randomize the edges by swapping (Supplementary Fig. 1b). Multiple interactions (parallel edges) and self-regulations (originating and ending at the same gene/operon) are not allowed, as in the original network.

The number of swaps required to create a random graph and the number of random graphs are determined by comparing the results of various combinations. We varied the number of null model graphs between 100 and 4000 checking for the robustness of our results. Results are shown for 1000 random graphs. We use the edge swap algorithm also to create randomized undirected CRNs. In this method, we randomize the edges of the original CRN by swapping. This method is denoted as “Edge Swap Random CRN" in the Results. The second method employed to generate a randomized CRN has two steps. First, we generate a randomized TRN using the edge swap method, and second, we build the CRN of the randomized TRN. This null model acknowledges the TRN as the ’hardware’ from which the relational structure, the CRN, is derived. The arguments in favor of this null model are similar to those motivating randomization of metabolite-centric metabolic networks on the level of the bipartite (metabolite-reaction) graph (see ref. ^49^).

As an additional consistency check for our data analysis pipeline, we create a randomized TRN (referred to in the following as ’base model’) and perform all analysis steps on this network, with the expectation that no nonrandom features will be discovered. Specifically, we generate randomized TRNs using the *n*^*th*^ generated network as the input (reference) network to generate the (*n*+1)^*st*^ network. Then, we build the CRN of each randomized TRN to check the consistency of the randomized CRNs. The aim is to test whether employing randomized networks as a base model affects the difference between the original and the randomized networks’ wiring lengths. If the randomization model is biased, the average total wiring length of the generated graphs are expected to be significantly different than the generated ones observed in Fig. 3. However, both generated random TRNs and CRNs show similar wiring length distributions with the ones observed in Fig. 3 with z-scores − 15.40 on TRN level and − 33.83 on CRN level (Supplementary Fig. 5).

The second method employed to generate random networks is the node swapping method. Two nodes (genes or operons, according to the level of TRN) are selected randomly, and the positions of the selected nodes are swapped. We perform on average 100 swaps per node. In this method, the set of node positions, as well as the interactions between the nodes, are preserved, i.e., the source and target nodes of the edges remain the same (Supplementary Fig. 1c). Randomized CRNs are constructed based on the randomized TRNs generated by node swapping.

In the random node position case, we assign random chromosomal positions to all nodes (genes/ operons) without altering the interactions between nodes. As in the node swapping case, the source and target nodes of the edges remain the same (Supplementary Fig. 1d). Randomized CRNs are constructed based on the randomized TRNs generated using the random node position method.

### Gene expression data

We use the Precise dataset, which offers 278 high-quality E. coli RNA-seq expression profiling datasets from over 15 studies. The dataset contains 20% of RNA-seq datasets available in NCBI GEO33 for E. coli K-12 MG1655 and BW25113^36^. RNA-seq provides a time-averaged and population-averaged estimate of the mRNA level of each gene.

## Supplementary information

Supplementary Information

## Data Availability

All data analysed during this study are publicly available via the databases referenced in the article.

## Code availability

The code used to perform the analyses presented in the current study is available from the corresponding author on reasonable request.
