# Supplementary material for: The economy of chromosomal distances in bacterial gene regulation
Source: NPJ Syst Biol Appl. 2021 Dec 15;7:49. doi: 10.1038/s41540-021-00209-2 (PMC8674286; doi:10.1038/s41540-021-00209-2)
Supplement: Supplementary file 1 — Supplementary Information [file 41540_2021_209_MOESM1_ESM.pdf]

Supplementary Information

Supplementary Text 1: Relating wiring length and expression level of the source gene

Starting point

We investigate the relationship between the expression level X_i of the TF i, measured using RNA-seq, and the average/total wiring length of the node i (source gene encoding the TF i).

Our main assumption is that the mechanistically relevant quantity is the local and instantaneous number x_i of newly synthesized TFs at the source locus i (translation being co-localized with transcription in bacteria). What is measured is the expression level X_i obtained by RNA-seq, i.e. an average over time, space and millions of cells. The two quantities are presumably proportional: $X_i = C_i x_i$, where the multiplicative factor C_i may depend on the TF i. We cannot infer the value of C_i so the only choice is to assume a constant factor C. For the basic considerations presented here, we will thus write $X_i = Cx_i$ with the same constant factor C for all source loci i. Deviations from this approximation can be expected to account for part of the noise in Figure 4 in the main text.

The second assumption is first-order kinetics, yielding an exponential decrease $e^{-K_i t}$ of TF concentration along time. Presumably there is no degradation during 1D sliding (fine-tuning of the search process, passing from weak non-specific DNA binding to a strong binding to a specific recognition site), hence only the 3D search time has to be taken into account in quantifying TF proteolysis. The possibility of 1D sliding also decreases the dilution effect (irreversible loss of TFs) however by a roughly homogeneous factor. Overall, we will ignore the contribution of 1D sliding in this basic investigation of the relationship between x_i and wiring length w_i.

We thus investigate the relationship between $X_i \approx Cx_i$ and the average wiring length w_i (distance in space between the source and target nodes, averaged over the target nodes). The initial TF amount x_i experiences both dilution (TFs not reaching the target in a sufficiently short time) and degradation (exponential dependence on the reaching time $T(w_i)$). In order that a proper regulation is achieved, the amount x_i has to be increased to compensate both the degradation loss (factor $e^{K_i T(w_i)}$) and the dilution loss.

For a simple deterministic flux, the dilution loss would be proportional to the surface πw_i^2 of the sphere,

so that the prediction becomes:

$$X(w) \sim Cw^2 e^{KT(w)}.$$

In order to simplify the problem further, we assume that the degradation kinetics occurs at the same scale of all TFs, i.e. the order of magnitude of K_i does not depend on i, and the dependence of K_i on i could be ignored. We then need an estimate of the reaching time $T(w)$.

Three options for estimating the reaching time $T(w)$

Our first approach is the naive estimate of a characteristic time based on the (possibly anomalous) diffusion law $w^2 \approx \langle r(t)^2 \rangle \sim At^\alpha$ (where $\alpha = 1$ in case of normal diffusion), yielding $T(w) \sim w^{2\alpha}$. The relationship would then be $X(w) \sim C\pi w^2 e^{K'w^{2\alpha}}$ (with $K' = K/A^{2/\alpha}$), i.e. $\ln X \sim 2\ln w + K'w^{2\alpha} + \ln C$, with different behaviors of $\ln X$ at small wiring lengths ($\ln w$ dominates) and large wiring length ($K'w^{2\alpha}$ dominates).

A second possibility is the full computation of the mean first passage time. Here it is unclear whether we should consider a motion in a confined space or not. A general behavior for normal diffusion in a (rectangular 3D) confined space is that the reaching time saturates at large distance, i.e. $T(w)$ tends to T_∞ at large w. The behavior at large wiring lengths would then be $X(w) \sim w^2$.

As a third possibility, one can make use of the calculations of Pulkinnen and Metzler[1]. They provide an expression, Eq. (8) in[1], of the local concentration $\Phi(t|r)$ of the TF in a neighborhood of its target gene, given that the TF experienced a transcriptional burst at $t = 0$ and that the distance between the TF and the target gene is r (i.e. $r = w$). This concentration displays a maximum along time, at a time $T^*(r)$ increasing with r, which provides a plausible expression for the reaching time T.

It should be emphasized again that any test of such detailed predictions of $T(w)$ and the scaling of x_i with w_i would require gene expression data beyond the currently available RNA-seq averages.

References

1. Pulkkinen, O. & Metzler, R. Distance matters: the impact of gene proximity in bacterial gene regulation. *Phys. Rev. Lett.* **110**, 198101 (2013).

Supplementary Table 1. Spearman correlation coefficients and corresponding p-values comparing gene expression profiles with various structural metrics of the gene-level TRN. Correlations and p-values are averages over all 278 gene expression profiles.

Measure	Average correlation	Average p-value
Total wiring length	0.416505	1.30977×10^{-6}
Average wiring length	0.336898	1.09819×10^{-4}
Out-degree	0.365207	2.21431×10^{-5}
Maximal wiring length	0.403927	1.78997×10^{-6}

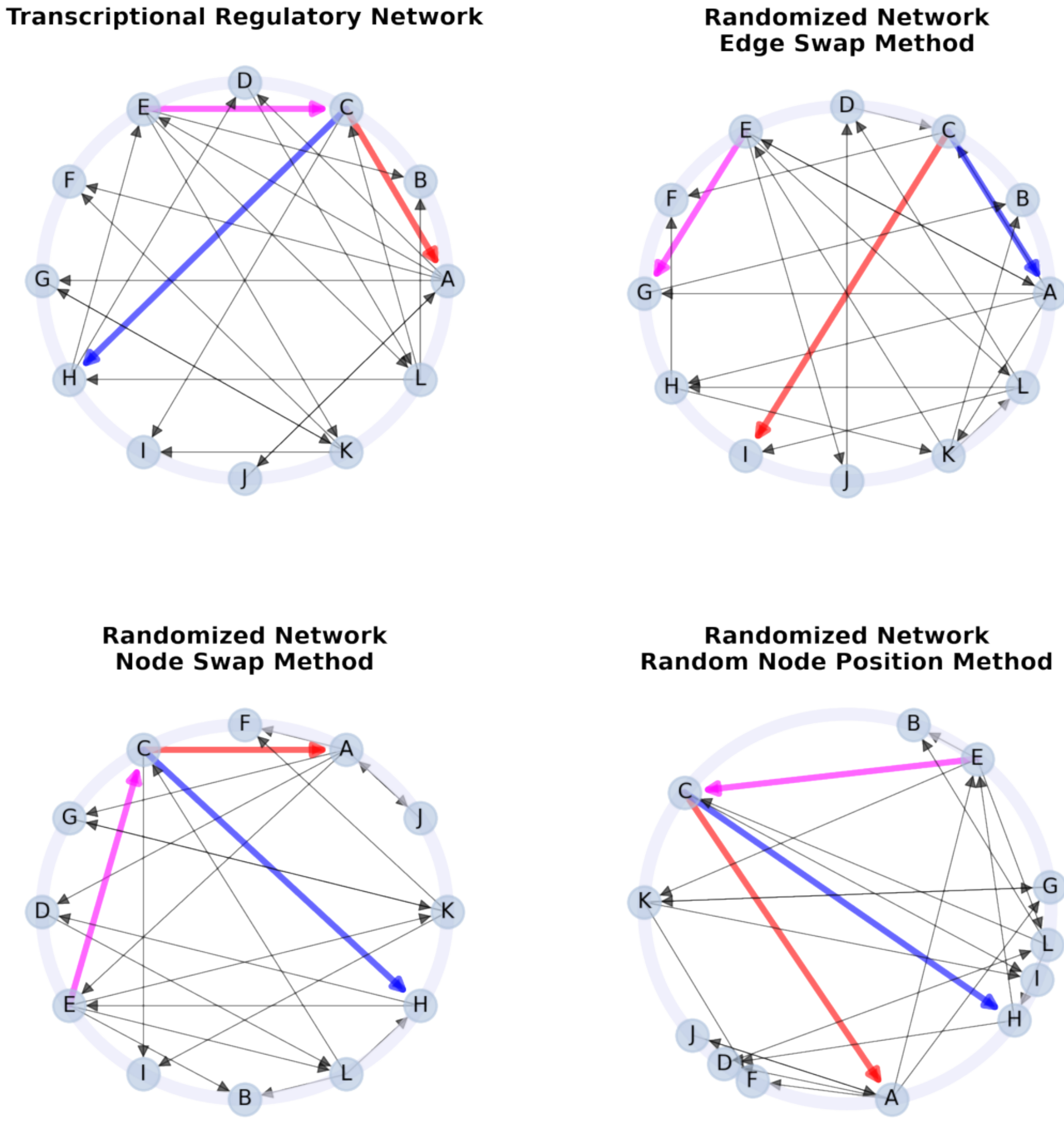

Supplementary Figure 1. Schematic illustration of the different null models employed in our study. (a) Schematic representation of the original TRN (same as Fig. 2a in the main text) for reference. Three edges are highlighted. The position of the three edges can be tracked in the randomized graphs. (b) Randomized network generated using the edge swap method. While node positions and source nodes of the edges are preserved, target nodes are randomized. (c) Randomized network generated using the node swap method. The nodes are randomly positioned within the set of original node locations. The source and target nodes of the edges remain the same as in the original network. (d) Randomized network generated using the random node position method. Random chromosomal positions are assigned to the nodes with no double occupancy. The original list of the node positions is not preserved. The source and target nodes of the edges remain the same as in the original network.

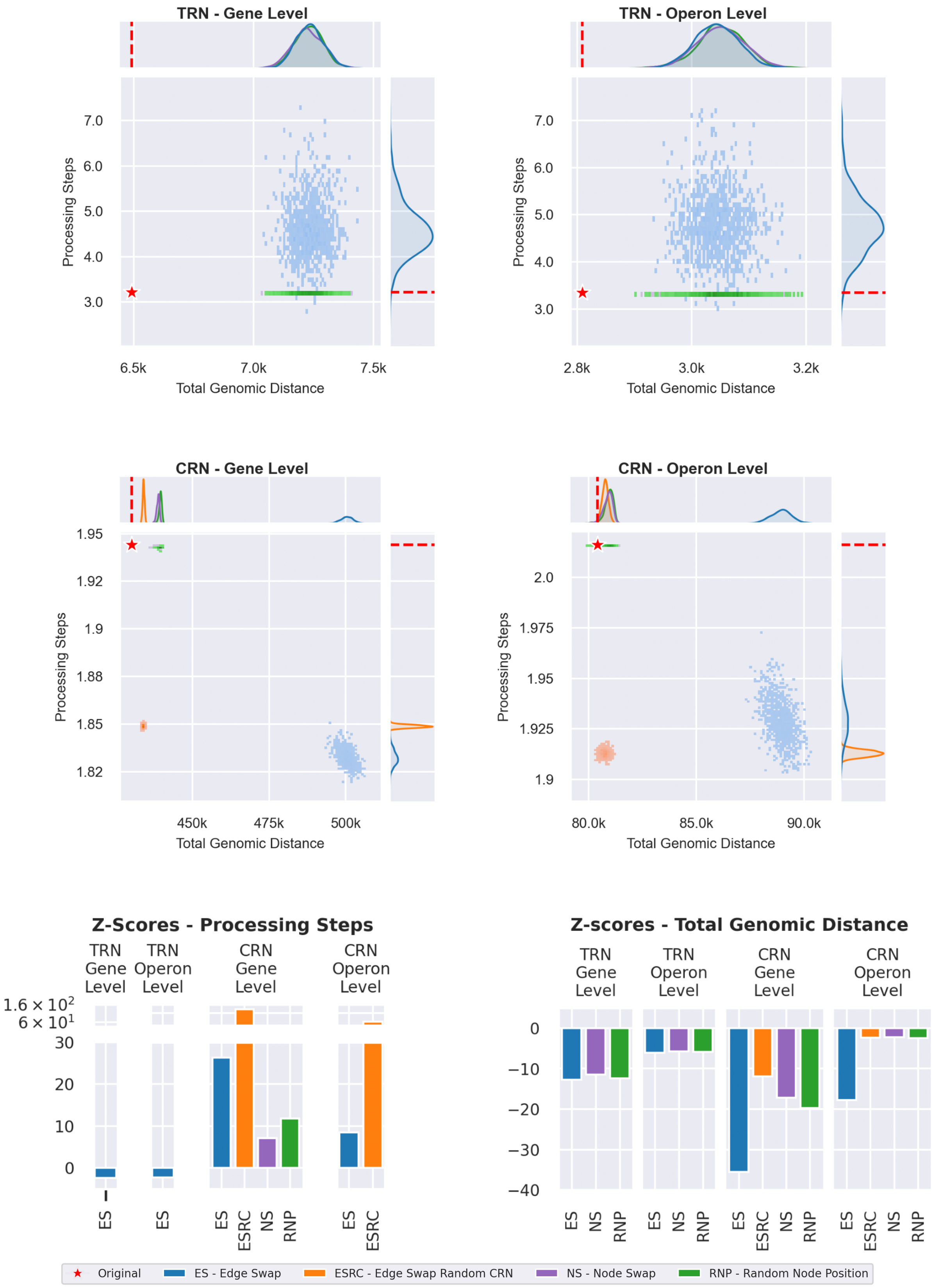

Supplementary Figure 2. Same as Figure 3 in the main text, but for genomic distances instead of wiring lengths.

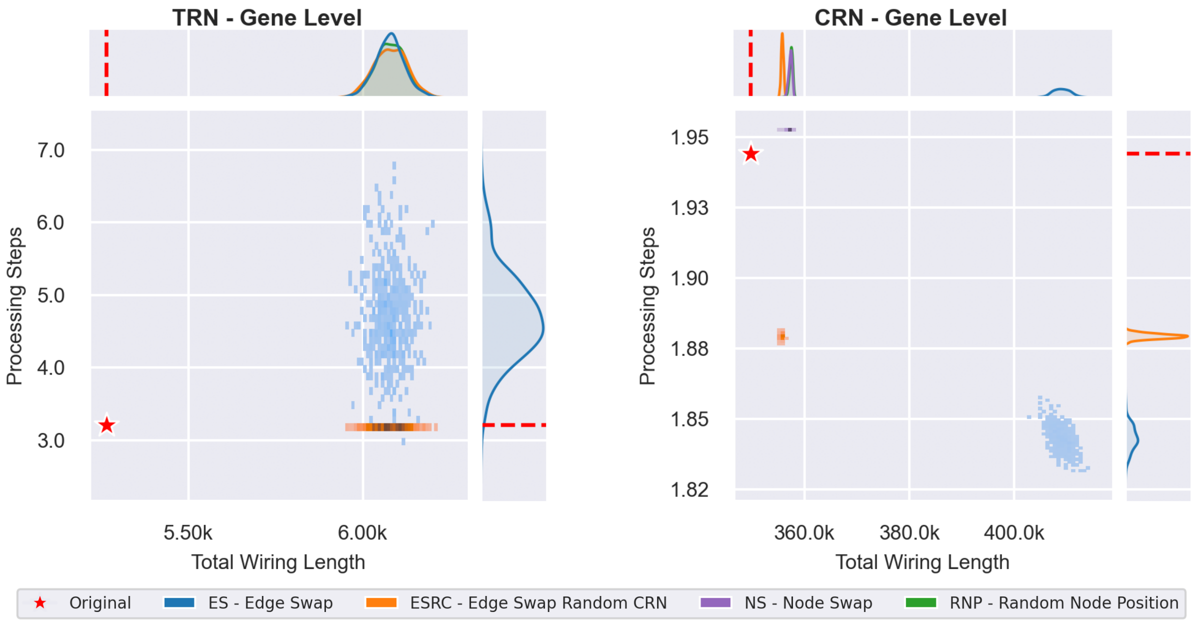

Supplementary Figure 3. Comparison of the original network and null models in terms of wiring length and number of processing steps on gene level without eliminating edges within the same operon. 500 randomized networks are generated for each null model.

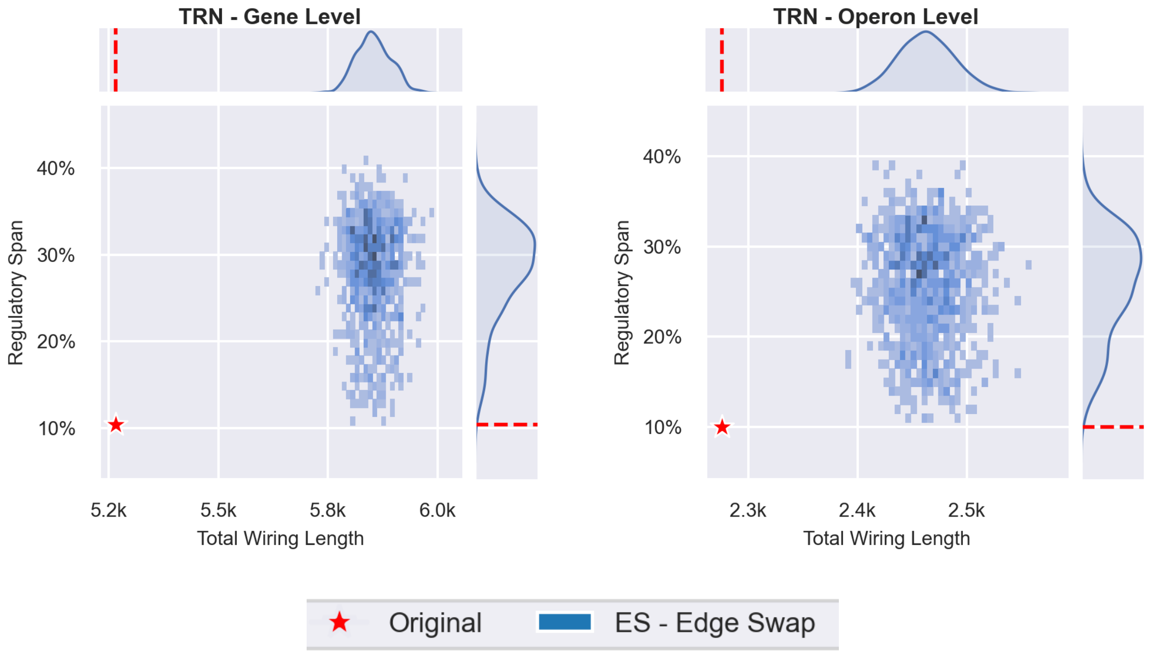

Supplementary Figure 4. Regulatory Span. The percentage of reachable nodes by some consistent path from a source node in comparison to the total number of nodes in a network is also evaluated. While 28.2% of the nodes are reachable on average on gene level at randomized network, the index remains at 10.4% at the original TRN.

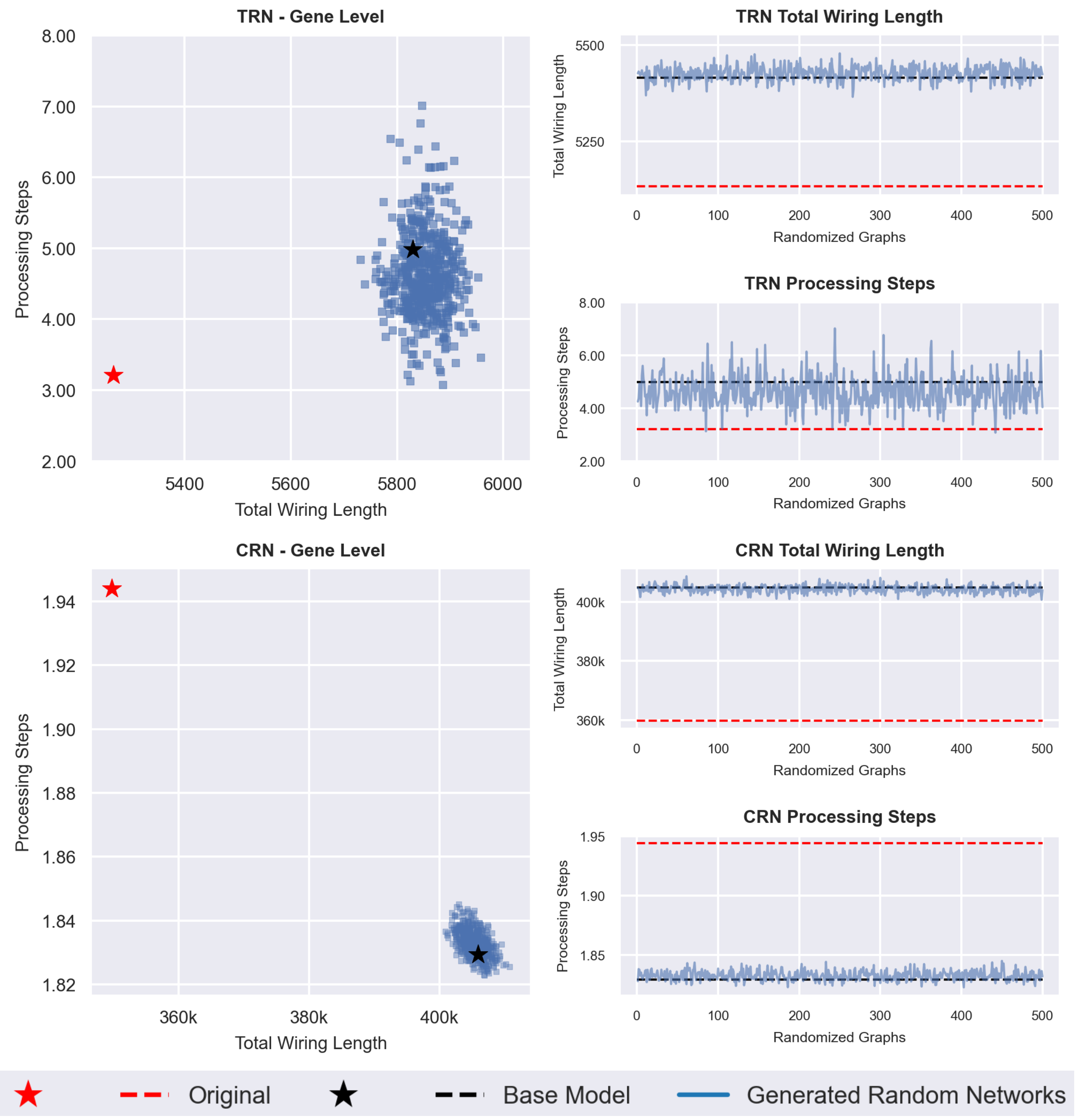

Supplementary Figure 5. Edge Swap Method Consistency Check. We assess the quality of our randomization methods by selecting one of the randomized networks as a reference network (or 'base model') and then contrast it with its randomized versions. We generate randomized TRNs using the nth generated network as the input (reference) network to generate the $(n+1)^{st}$ network. Then, we build CRN of each randomized TRN to check the consistency of the randomized CRNs. Both generated random TRNs and CRNs show similar wiring length distributions with the ones observed in Figure 3 in the main text with z-scores -15.40 on TRN level and -33.83 on CRN level.
